# Long non-coding RNA maternally expressed gene 3, miR-125a-5p, CXCL13, and NF-kB in patients with immune thrombocytopenia

**DOI:** 10.1038/s41435-023-00200-3

**Published:** 2023-04-12

**Authors:** Mervat Naguib, Shereen El Sawy, Laila Rashed, Maha AlHelf, Marwa Abdelgwad

**Affiliations:** 1 Internal Medicine Department, Faculty of Medicine Kasr Al-Ainy Hospital, Cairo University, Cairo, Egypt; 2 Medical Biochemistry and Molecular Biology Department, Faculty of Medicine, Cairo University, Cairo, Egypt; 3 Biotechnology School, Nile University, Giza, Egypt

**Keywords:** Genetics, Immunogenetics

## Abstract

The main aim of this study was to assess the expression level of circulating long non-coding RNA maternally expressed gene 3 (lncRNA-MEG3), microRNA (miR-125a-5P), the chemokine C-X-C motif ligand13 (CXCL13), and the nuclear factor kappa-light-chain-enhancer of activated B cells (NF-kB) in immune thrombocytopenia (ITP) cases and to study its relation to the disease severity and treatment response. This case-control study included 45 patients newly diagnosed as ITP and 45 healthy subjects. We assessed complete blood count, antinuclear antibodies, hepatitis B and C virus serology, lncRNA-MEG3, miR-125a-5P, and CXCL13 expression in serum by real-time PCR and NF-kb protein by ELISA. In ITP patients compared to control, lncRNA-MEG3 was significantly increased, and miRNA-125a-5P was decreased, and this was associated with higher CXCL13 and NF-kB levels (*P* < *0.001*, for all).There was a significant negative correlation between platelet count and lncRNA-MEG3, CXCL13, and NF-kb, while a positive correlation with miR-125a-5p in ITP patients. Patients who responded to steroids had significantly higher miR-125a-5p (*P* = *0.016*) and significantly lower lncRNA-MEG3 (*P* < *0.001*), CXCL13 (*P* = *0.005*), and NF-kb (*p* = *0.002)*. Based on the ROC curves, lncRNA-MEG3 displayed the highest area under the curve (AUC) in the identification of organ bleeding (AUC = 0.805), the response to steroids (AUC = 0.853), and the need for splenectomy (AUC = 0.75).

## Introduction

Primary immune thrombocytopenia (ITP) is a common autoimmune disease characterized by immune-mediated increased platelet destruction and impaired platelet assembly [1]. The pathogenesis of ITP is multifactorial, involving both the disturbed function of B and T lymphocytes [2]. Cluster of differentiation antigen 4 (CD4+) T cells subclasses, including; helper T cells (Th1), (Th2), (Th17), and regulatory T cell (Treg), are the main subclasses implicated in regulating the immune processes and cytokine profiles. Consequently, these T-cells have a significant role in different immune diseases [3]. There is increasing evidence that subclasses of T cells contribute to the development and progression of ITP; however, the implicated molecular mechanisms are unclear [4].

Patients with ITP have reduced Treg number and or function [4]. Micro-ribonucleic acid (miRNA) and long non-coding RNA (lncRNA) have recently been detected to have a crucial effect in the immunosuppressive function of Tregs through their effect on gene expression at the post-transcriptional stage [5]. miRNAs are short (~18–25 nucleotides long), non-coding RNAs that control gene expression at the post-transcriptional stage, through epigenetic change, and have an established effect on the immune cells maturation, differentiation, and function [6]. LncRNAs are highly structured RNA transcripts longer than 200 nucleotides that are not translated to proteins and have been linked to multiple human diseases through the regulation miRNAs and genes they target [6].

About 37 miRNAs expressed significantly in patients with ITP, there are 26 miRNAs that are upregulated and 11 miRNAs that are downregulated. ITP patients have downregulated miR-125a-5p levels in Tregs, which contributes to Treg dysfunction and ITP pathogenesis [7].

Long non-coding RNA maternally expressed gene 3 (lncRNA-MEG3) is commonly reported in tumor biology, and its role in ITP needs further clarification [8]. One study showed that lncRNA-MEG3 in CD4 + T cells was the highest differentially expressed lncRNA between patients with ITP and healthy controls [9]. It has also demonstrated that lncRNAs are involved in the regulation of miRNA expression [10], and lncRNA-MEG3 might directly interact with miR-125a-5p [11].

Chemokine C-X-C motif ligand13 (CXCL13) is a small cytokine involved in multiple immune responses through chemoattraction and activation of leukocytes [12]. It has been reported that CXCL13 plays a vital role in many immune diseases through the recruitment of different T-cell subtypes and is a promising therapeutic target in immune diseases [13]. Some studies suggested a possible role of CXCL13 in the pathogenesis of ITP [9].

Nuclear factor-kappa B (NF-κB) is a transcription complex crucial for host defense and is mediated by innate and adaptive immunity [14]. Defects in NF-κB contribute to autoimmune diseases, immune deficiency and inflammatory diseases, and lymphoid malignancy [14]. There is also abundant evidence that NF-κB is necessary for maintaining immune tolerance due to its actions during thymic selection, both for negative selection of autoreactive T cells and selection and maintenance of Tregs [14]. Yu et al. reported that increased activation of NF-κB may promote the development of ITP by the NOD-like receptor pyrin domain-containing protein 3 (NLRP3) inflammasome [15].

Few studies have discussed the relationship between levels of lncRNA-MEG3, miR125a-5P, and CXCL13, in patients with ITP [9, 16]; moreover, NF-kB involved in Treg cell function needs further evaluation in this group of patients. One study reported that LncRNA-MEG3 induced an imbalance in the Treg/Th17 ratio through inhibiting miR-125a-5p; which then enhanced Th17-cell differentiation and led to an immune imbalance in ITP patients [9]. Another study found that CXCL13 was the target gene of miR-125-5p and MiR-125-5p inducer decreased CXCL13 level and miR-125-5p inhibitor elevated CXCL13 level in CD4 + T cells [16]. Also, lncRNA-MEG3 has recently been discovered to activate NF-κB pathway by miRNA [17]. Furthermore, the role of miR-125a in suppressing tumor necrosis factor alpha-induced protein 3 (TNFAIP3) and aberrantly activating the NF-κB pathway has been reported [18].

From the above-mentioned data, it seems that lncRNA-MEG3, miR-125a-5P, CXCL13 and NF-kB play an important role in the pathogenesis of ITP. It is still unclear how these RNAs and circulating proteins correlate to each other and to the severity of the disease in ITP patients. Furthermore, few in vitro studies reported the effect of Dexamethasone on these RNAs and circulating proteins in patients with ITP [9, 16]. The current study aimed to determine the serum level of circulating lncRNA-MEG3, miR-125a-5P, CXCL13, and NF-kB in patients with ITP compared to healthy subjects. In addition, the relation of these markers to each other, the disease severity, and treatment response were investigated.

## Material and methods

### Study design

This study was a prospective case-control study carried out in a tertiary care referral hospital.

### Ethical approval

The study has been approved by ethical committee of Kasr Al -Ainy Hospital, Faculty of Medicine, Cairo University. Informed consent was obtained from all participants. The study protocol and procedures conform to the ethical guidelines of the 1975 declaration of Helsinki.

### Sample size

Sample size has been calculated based on assumptions from pilot study over to patients conducted by the researcher with AUC of CLXC 13 in predicting the steroid response as primary outcome for this research using Med calc. for sample size based on AUC value of study parameter where: two sided alpha of 0.05, power of 0.9 and AUC were 0.95 and null hypothesis AUC 0.6 and the ratio of positive to negative group 1:1. The minimum required number was 16 patients. After adding 20% for considering drop out so estimated sample size will be 20 patients.

### Subjects and methods

This study included 90 subjects, 45 adults with ITP and 45 healthy subjects as controls. Before starting treatments, adult patients newly diagnosed with ITP were recruited from outpatient clinics or those admitted to the hospital. Patients with (1) Secondary ITP; 2ry to HCV, autoimmune disease, thyroid disease or drug-induced, (2) Chronic ITP, (3) Autoimmune diseases, (4) Malignancy, and (5) Liver cirrhosis or chronic renal disease were excluded. Collected clinical data included age, gender, thorough medical history, physical examination, medications, bleeding site, and severity.

Blood samples were drawn from each participant for complete blood count (CBC), peripheral blood film, antinuclear antibodies (ANA), hepatitis C virus (HCV) serology, and hepatitis B virus (HBV) serology. LncRNA-MEG3, miR-125a-5P, and CXCL13 gene expressions in serum from ITP patients and healthy subjects were measured by real-time polymerase chain reaction (PCR). Enzyme-linked immunoassay (ELISA) was used to detect NF-kB level.

Patients were classified according to the site of bleeding into three groups; (1) skin, (2) mucosal, and (3) Organs [19]. Also, the patients were classified according to the need for transfusion into two groups; (1) Patients who required transfusion and (2) Patients who did not need a transfusion. Patients were treated based on the updated international consensus report on the investigation and management of primary immune thrombocytopenia [20].

### Biochemical assay

#### Blood sample collection and storage

For each patient, 3 ml peripheral blood sample was collected from each subject and placed in plain tube for centrifugation and serum separation. After that, serum was kept at −80 °C for total RNA with preserved micro-RNA extraction and real-time PCR quantification of the fold changes of miR-125a-5P gene as well as lncRNA-MEG3, and CXCL13 mRNAs. In addition, the serum was also used to estimate the serum level of NF-κB. The investigator was blinded to the group allocation during the experiment and when assessing the outcome.

#### Measurement of LncRNA-MEG3, miR-125a-5P, and CXCL13 fold changes

##### A-Isolation of RNA

From 200 μL of serum, total RNA was isolated along with preserved micro-RNAs by miRNeasy extraction kit (Qiagen, Valencia, CA, USA). The isolation procedure was done as follow; (1) - One mL QIAzol lysis reagent was added to 200 μL serum and incubated for 5 min at room temperature. (2) - Two hundred μL chloroform was added. (3) - The mixture was shaken vigorously using vortex for 15 s. (4) - Incubation was done for 2–3 min at room temperature. (5) - Centrifugation at 12000 × *g* at 4 °C was done for 15 min. (6) - The upper watery phase was removed, and 1.5 times of its volume 100% ethanol was added. (7) - Seven hundred uL of this mixture was placed in RNeasy Mini spin column in 2 ml collection tube and centrifuged at 8000 × *g* at room temperature for 15 s. (8) - After the mixture had completely passed the column, 700 μL of buffer RWT was added to each column, and again centrifuged at 8000 × *g* at room temperature for 15 s. (9) - Five hundred μL buffer RPE was added to the column and centrifuged at 8000 × *g* at room temperature for 15 s. (10) - The previous process was repeated with centrifugation for 2 min at full speed(14,000 × *g*). (11) - The column was transferred to new 1.5 ml collection tube and 50 uL RNase- free water was pipetted directly onto the column and centrifuged for 1 min at 8000 × *g* to elute RNA.

RNA samples were subjected to RNA quantitation and purity assessment using the NanoDrop® (ND)-1000 spectrophotometer (NanoDrop Technologies, Inc. Wilmington, USA). Steps of the procedure were as follow: (1) -The Nano-Drop spectrophotometer was blanked, a spectrum was taken of a reference material (blank) and stored in memory as an array of light intensities by wavelength. (2) - A measurement of a sample was taken; the intensity of light that had transmitted through the sample was recorded. (3) - The samples intensities along with the blank intensities were used to calculate the sample absorbance according to the following equation: Absorbance = −log (Intensity sample /Intensity blank). The calculation of the concentration was automated [21–23].

##### B-Reverse transcription (RT) and real-time quantitative PCR (qPCR)

**LncRNA-MEG3 and CXCL13 quantification**

Total RNA (0.5–2 μg) was utilized for complementary double stranded nucleic acid (cDNA) conversion using a high-capacity cDNA reverse transcription kit (Fermentas, USA). The fold changes of lncRNA-MEG3 and CXCL13 were quantified through Real-time qPCR amplification and analysis using an Applied Biosystem with software version 3.1 (StepOne™, USA). The qPCR assay with the primer sets were optimized at the annealing temperature. GAPDH was utilized as a housekeeping gene.

The sequences of the amplification primers were as follows:

- lncRNA-MEG3 forward 5’-CTGCCCATCTACACCTCACG-3′; reverse 5′-CTCTCCGCCGTCTGCGCTAGGGGCT-3′
- CXCL13 forward 5′-GAGGCAGATGGAACTTGAGC-3′; reverse 5′-CTGGGGATCTTCGAATGCTA-3′
- GAPDH forward 5′-GAAGGTGAAGGTCGGAGTCA-3′; reverse 5′-GAAGATGGTGATGGGATTTC-3′.

Melting curve analyses were performed following the PCR cycles to confirm the precise production of the anticipated PCR product. The results were interpreted using the threshold cycle (2^−ΔΔCt^) method of comparative PCR.

##### miR-125a-5P quantification

According to the manufacturer’s protocol using the miScript II RT kit (Qiagen, Valencia, CA, USA), extracted RNA (1 μg) was reverse transcribed to cDNA in a final volume of 20 μL RT reactions (incubated for 60 min at 37 °C, followed by 5 min at 95 °C) for miR-125a-5P PCR quantification.

Real-time quantitative PCR test was performed using miScript SYBR green PCR kit (Qiagen) that contained miR-125a-5P-specific forward primer (5′-CCTGAGACCCTTTAACC-3′) and its reverse primer (5′-GAACATGTCTGCGTATCTC-3′) in addition to U6 as an internal reference (forward primer; 5′GCTTCGGCAGCACATATACTAAAAT-3′ and reverse primer; 5′-CGCTTCACGAATTTGCGTGTCAT-3′).

The steps for reverse transcription and cDNA synthesis were as follows: (1) - the reverse-transcription master mix was prepared on ice. (2) - Template RNA was added to each tube containing reverse-transcription master mix. Gently mixing was done with centrifugation. (3) - Incubation was done for 60 min at 37 °C. (4) - Incubation for 5 min at 95 °C was done to inactivate miScript Reverse Transcriptase (for miR-125a-5P) and the high-capacity cDNA reverse Transcriptase in case of lncRNA-MEG3 and CXCL13.

The steps for qPCR tests were as follows: (1) - Reaction mix was prepared for a 25 μl per well reaction volume. (2) - The real-time cycler was programmed as follows: (a)-PCR Initial activation step at 95 °C for 15 min. (b) Three-step Cycling: denaturation at 94 °C for 15 s, annealing at 55 °C for 30 s and extension at 70 °C for 30 s.

#### Serum NF-κB measurement

Human NF-κB ELISA Kit, Catalog No. MBS450580, a sandwich enzyme immunoassay, was used for the in vitro quantitative detection of NF-κB in serum. All kit reagents and standards were prepared according to the manufacturer’s instructions. The color change was measured spectrophotometrically at a wavelength of 450 nm ± 10 nm. The concentration of NF-κB in the samples was then determined by comparing the samples’ optical density measurement (OD) to the standard curve. The assay procedure was done as follows: (1) - All reagents, samples and standards were prepared. (2) - 100 µL standard or sample was added to each well. Incubation for 2 h at 37 °C was done. (3)- 100 µL prepared Detection Reagent A was aspirated and added. Incubation for 1 h at 37 °C was followed. (4) - Aspiration and washing was done for successive 3 times. (5) - 100 µL prepared Detection Reagent B was then added followed by incubation for 1 h at 37 °C. (6) - Aspiration and washing for 5 times was done and (7) - 90 µL Substrate Solution was added followed by incubation 15–25 min at 37 °C. (8) - Finally, 50 µL Stop Solution was added. The readings were detected at 450 nm immediately [24].

### Statistical analysis

Data was entered and statistically analyzed on the Statistical Package of Social Science Software program, version 23 (IBM SPSS Statistics for Windows, Version 23.0. Armonk, NY: IBM Corp.). Data were presented using mean and standard deviation for normal data and frequency and percentage for qualitative ones. Comparison between groups for qualitative variables was performed using Chi-square or Fisher’s exact test when one expected cell or more is <5. For quantitative variables, the comparison was conducted using sample *t*-test (for parametric data) and a Mann–Whitney test (for non-parametric data). Spearman correlation test was used to assess the linear relationship between variables. Receiver Operating Characteristics (ROC) curve analysis was performed using Stata 16 to assess the discriminant ability of lncRNA-MEG3, miR-125a-5P, CXCL13, and NF-kB as predictors of the site of bleeding and steroid response. Statistical significance was defined as a *P* value less than or equal to 0.05.

## Results

### Clinical and laboratory parameters of patients with ITP compared with healthy controls

Forty-five patients with ITP and 45 healthy subjects were included in this study. The mean age of ITP patients was 34.58 ± 12.43 years, and about 84% were female, with no statistically significant difference between patients and controls. The mean platelet count in patients with ITP before starting treatment was 13.77 ± 12.24(×10^9^/L) and 123.96 ± 91.86(×10^9^/L) after treatment. ITP patients had significantly lower hemoglobin and platelet compared to control subjects.

In patients with ITP, ten patients (22.2%) had only cutaneous bleeding, 25 patients (55.6%) had visible mucosal bleeding, and ten patients (22.2%) had organ bleeding. Twenty-nine (64.4%) patients with ITP needed a transfusion, 44.4% responded to steroids, 24.4% needed treatment with Rituximab, and 13.3% had undergone splenectomy.

Patients with ITP compared to control subjects had significantly higher lncRNA-MEG3 (6.79 ± 1.98 vs.1.04 ± 0.07; *P* < *0.001)*, CXCL13 (115.22 ± 27.19 vs.6.75 ± 4.52 pg/mL;*P* < *0.001*), NF-kB (193.75 ± 38.63 vs.102.76 ± 9.7 pg/mL;*P* < *0.001*) but lower level of miR-125a-5p (0.59 ± 0.2 vs.1.03 ± 0.04;*P* < *0.001*).Table 1 shows comparison of clinical and laboratorial characteristics of patients and control.

**Table 1:** Comparison of clinical and laboratorial characteristics of patients with ITP and control.

Variable	Patients with ITP *n* = 45	Control subjects *n* = 45	^a^*P value*
Age (mean/years)	34.58 ± 12.43	34.51 ± 12.15	0.98
Gender (female/male)	38/7	39/6	0.58
Platelet count (×10^9^/L)	13.77 ± 12.24	364.18 ± 64.09	<0.001
Site of bleeding (No)%			
Skin	10 (22.2%)		
Mucosal	25 (55.6%)		
Organ	10 (22.2%)		
Patients received platelet transfusion	29 (64.4%)		
Patients needed Splenectomy	6 (13.3%)		
Patient responded to steroids	20 (44.4%)		
Patients needed Rituximab	11 (24.4%)		
^b^lncRNA-MEG3	6.79 ± 1.98	1.04 ± 0.07	<0.001
^c^miR-125a-5p	0.59 ± 0.2	1.03 ± 0.04	<0.001
^d^CXCL13(pg/mL)	115.22 ± 27.19	6.75 ± 4.52	<0.001
^e^NF-kB(pg/mL)	193.75 ± 38.63	102.76 ± 9.7	<0.001

^a^*P* value < 0.05 is considered significant.

^b^lncRNA-MEG3: long non-coding RNA maternally expressed gene 3.

^c^miR-125a-5p: microRNA-125a-5p.

^d^CXCL13: chemokine C-X-C motif ligand 13.

^e^NF-kB: the nuclear factor kappa-light-chain-enhancer of activated B cells.

### Relation of lncRNA-MEG3 level with different clinical and laboratory data in patients with ITP

Patients with organ bleeding had a significantly higher level of lncRNA-MEG3 than those with only skin bleeding *(P* = *0.024)*. lncRNA-MEG3 was significantly lower in ITP patients who responded to steroids than in non-responder *(P* < *0.001*). At the same time, It was significantly higher in patients who needed transfusion (*P* = *0.033)* and those who had a splenectomy *(P* = *0.049)* compared to those who did not (Table 2). lncRNA-MEG3 was negatively correlated to hemoglobin concentration (*r* = −0.452;*P* < *0.001*), platelet count (*r* = −0.764; *P* < *0.001*), and miR-125a-5p (*r* = −0.911; *P* < *0.001*), but positively correlated to CXCL13 (*r* = 0.884; *P* < *0.001*) and NF-kB (*r* = 0.822; *P* < *0.001*) (Table 3).

**Table 2:** lncRNA-MEG3, miR125a-5P, CXCL13 and NF-kB levels in patients with ITP.

Variable	^b^lncRNA-MEG3	^c^miR125a-5P	^d^CXCL13	^e^NF-kB (pg/mL)
Site of bleeding				
Skin	5.41 ± 1.94	0.72 ± 0.20	100.8 ± 24.56	172.52 ± 40.09
Mucosal	7.22 ± 1.91	0.53 ± 0.19	122.4 ± 28.31	202.09 ± 37.33
Organ	7.10 ± 1.69	0.6 ± 0.2	111.7 ± 22.04	194.13 ± 35.77
^a^*P value*	0.024	0.026	0.034	0.26
Platelet transfusion				
Yes	7.27 ± 1.85	0.53 ± 0.18	121.24 ± 25.63	202.74 ± 37.14
No	5.91 ± 1.95	0.68 ± 0.21	104.31 ± 27.3	177.46 ± 36.93
^a^*P value*	0.033	0.033	0.023	0.045
Steroid response				
Yes	5.35 ± 1.28	0.69 ± 0.15	98.94 ± 16.36	170.24 ± 28.55
No	7.66 ± 1.81	0.52 ± 0.21	125.11 ± 27.89	208.03 ± 37.28
^a^*P value*	<0.001	0.016	0.005	0.002
Need for Rituximab				
Yes	7.53 ± 1.54	0.55 ± 0.19	123.36 ± 24.82	204.85 ± 33.5
No	6.55 ± 2.06	0.60 ± 0.21	112.59 ± 27.74	190.16 ± 39.95
^a^*P value*	0.062	0.725	0.327	0.314
Need for splenectomy				
Yes	7.96 ± 1.93	0.53 ± 0.22	123 ± 31.65	213.67 ± 43.68
No	6.61 ± 1.94	0.6 ± 0.2	114.03 ± 26.7	190.69 ± 37.48
^a^*P value*	0.049	0.442	0.637	0.286

^a^*P* value < 0.05 is considered significant.

^b^lncRNA-MEG3: long non-coding RNA maternally expressed gene 3.

^c^miR-125a-5p: microRNA-125a-5p.

^d^CXCL13: chemokine C-X-C motif ligand 13.

^e^NF-kB: the nuclear factor kappa-light-chain-enhancer of activated B cells.

**Table 3:** Correlation of lncRNA-MEG3, miR125a-5P, CXCL13 and NF-kB levels with different variables in patients with ITP.

Variable	lncRNA-MEG3		miR-125a-5p		CXCL13 (pg/mL)		NF-kB (pg/mL)	
	*r*	^a^*P* value	*r*	^a^*P* value	*r*	^a^*P* value	*r*	^a^*P* value
Hemoglobin concentration	−0.452	<0.001	0.347	0.001	−0.374	<0.001	−0.433	<0.001
Platelet count	−0.764	<0.001	0.808	<0.001	−0.768	<0.001	−0.765	<0.001
Platelet at end of study	−0.294	0.05	0.182	0.232	−0.476	0.001	−0.374	0.011
lncRNA-MEG3			−0.911	<0.001	0.884	<0.001	0.822	<0.001
miR-125a-5p	−0.911	<0.001			−0.870	<0.001	−0.856	<0.001
CXCL13	0.884	<0.001	−0.870	<0.001			0.848	<0.001
NF-kB(pg/mL)	0.822	<0.001	−0.856	<0.001	0.848	<0.001		

^a^*P* value < 0.05 is considered significant.

### Relation of miR-125a-5p expression level with different clinical and laboratory data in patients with ITP

miR-125a-5p level was significantly lower in patients with organ bleeding compared to those with skin bleeding (0.6 ± 0.2 vs. 0.72 ± 0.2;*P* = *0.026*). miR-125a-5p was significantly increased in ITP patients who responded to steroids (*P* = *0.016*). Patients who needed transfusion had significantly lower miR-125a-5p levels than those who did not. ITP patients who underwent splenectomy or received Rituximab had no significant difference in miR-125a-5p expression level compared to those who did not (Table 2). miR-125a-5p had positive correlation to hemoglobin concentration (*r* = 0.347; *P* = *0.001*),and platelet count (*r* = 0.808; *P* < *0.001*), but negative correlation to CXCL13 (*r* = −0.870; *P* < *0.001*) and NF-kB (*r* = −0.856; *P* < *0.001*) (Table 3).

### Relation of CXCL13 level with different clinical and laboratory data in patients with ITP

Patients with organ bleeding had significantly higher CXCL13 levels than those with only skin bleeding *(P* = *0.034)*. CXCL13 level was significantly lower in ITP patients who responded to steroids compared to non-responders *(P* = *0.005*), and It was significantly higher in patients who needed transfusion compared to those who did not (121.24 ± 25.63 vs. 104.31 ± 27.3; *P* = *0.023*) (Table 2). CXCL13 level was negatively correlated to hemoglobin concentration (*r* = −0.374; *P* < *0.001*), platelet count (*r* = −0.768; *P* < *0.001*), and miR-125a-5p (*r* = −0.870; *P* < *0.001*), but positively correlated to lncRNA-MEG3 (*r* = 0.884; *P* < *0.001*) and NF-kB (*r* = 0.848; *P* < *0.001*) (Table 3).

### Relation of NF-kB level with different clinical and laboratory data in patients with ITP

NF-kB level was not significantly related to the bleeding site (*P* = 0.26). NF-kB level was significantly higher in ITP patients who did not respond to steroids compared to responders *(P* = *0.005*), and It was significantly higher in patients who needed transfusion compared to those who did not (202.74 ± 37.14 vs.177.46 ± 36.93; *P* = *0.045*) (Table 2). NF-kB level was negatively correlated to hemoglobin concentration (*r* = −0.433; *P* < *0.001*), platelet count (*r* = −0.765; *P* < *0.001*), and miR-125a-5p (*r* = −0.856; *P* < *0.001*), but positively correlated to lncRNA-MEG3 (*r* = 0.822; *P* < *0.001*) and CXCL13 (*r* = 0.848; *P* < *0.001*) (Table 3).

### ROC curves diagnostic test accuracy for the prediction of the need for transfusion, bleeding site and treatment response

The ROC curves diagnostic test accuracy of lncRNA-MEG3, miR-125a-5P, and CXCL13 were statistically significant discriminators of the occurrence of organ bleeding, with the highest area under the ROC curve (AUC) = 0.805 (95% CI 0.569–0.945) *(P* = *0.024)* for lncRNA-MEG3 followed by miR125a-5P (AUC) = 0.710 (95% CI 0.457–0.881) *(P* = *0.026)*. lncRNA-MEG3 has the highest (100%) sensitivity with 70% specificity for the prediction of organ bleeding (Table 4).

**Table 4:** lncRNA-MEG3, miR125a-5P, CXCL13 and NF-kB as predictors of site of bleeding and treatment response.

Predictors of organ bleeding							
	AUC	95% CI	Cut off	Sensitivity	Specificity	+PV	−PV
lncRNA-MEG3	0.805	0.569–0.945	>5.1	100	70	76.9	100
miR-125a-5p	0.710	0.457–0.881	≤0.84	100	50	66.7	100
CXCL13	0.690	0.447–0.874	>121	60	90	85.7	69.2
NF-kB(pg/mL)	0.660	0.418–0.853	>128.3	100	30	58.8	100
*Predictors of steroid response*							
lncRNA-MEG3	0.853	0.716–0.941	≤7.2	94.12	64.29	61.5	94.7
miR-125a-5p	0.716	0.562–0.841	>0.43	100	53.57	56.7	100
CXCL13(pg/mL)	0.749	0.597–0.866	≤121	88.24	67.86	62.5	90.5
NF-kB(pg/mL)	0.780	0.632–0.890	≤201.9	88.24	53.57	53.6	88.2
*Predictors of need for splenectomy*							
lncRNA-MEG3	0.750	0.599–0.867	>5.7	100	51.28	24	100
miR-125a-5p	0.603	0.446–0.745	≤0.42	66.67	74.36	28.6	93.5
CXCL13	0.564	0.408–0.711	>125	66.67	58.97	20	92
NF-kB(pg/mL)	0.639	0.482–0.777	>211.2	66.67	76.92	30.8	93.8

*AUC* area under of the curve, *CI* confidence interval, *PV* predictive value.

**Fig. 1:**
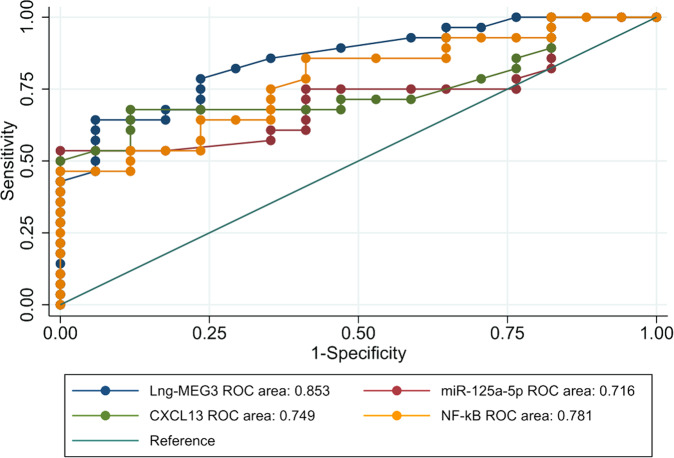
ROC curve diagnostic test accuracy of Lng- MEG3, miR125a-5P, CXCL13 and NF-kB for response to steroids in patients with ITP. ROC curve diagnostic test accuracy for response to steroids in patients with ITP showed lncRNA-MEG3 (AUC = 0.853), miR-125a-5p (AUC = 0.716), CXCL13 (AUC = 0.749) and NF-kB (AUC = 0.780).

A cutoff point of 7.2 for lncRNA-MEG3 at presentation could significantly define steroid responders and non-responders (AUC = 0.853; *P* ≤ *0.001*) with 94% sensitivity. Also, miR-125a-5P, CXCL13 and NF-kB levels before treatment were significant predictors of the response to steroids with (AUC = 0.716; *P* = *0.016*), (AUC = 0.749;*P* = *0.005*), (AUC = 0.780;*P* = *0.002*) respectively (Table 4) (Fig. 1)

**Fig. 2:**
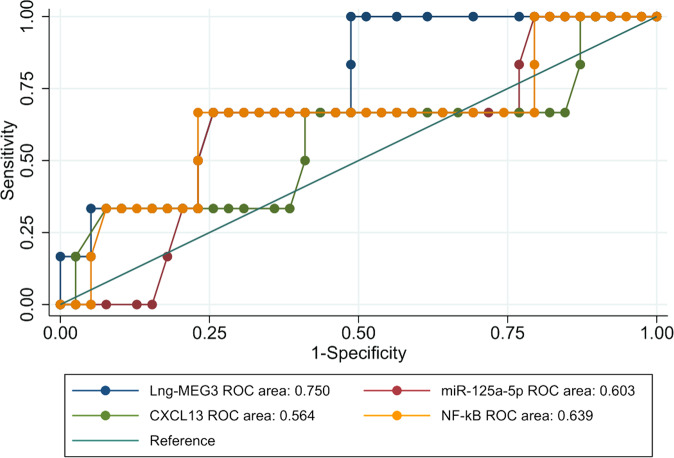
ROC curve diagnostic test accuracy of Lng- MEG3, miR125a-5P, CXCL13 and NF-kB for need to splenectomy in patients with ITP. ROC curve diagnostic test accuracy for need to splenectomy in patients with ITP showed lncRNA-MEG3 (AUC = 0.750), miR-125a-5p (AUC = 0.603), CXCL13 (AUC = 0.564) and NF-kB (AUC = 0.639).

ROC curves of lncRNA-MEG3, miR-125a-5P, CXCL13, and NF-kB for the need for splenectomy showed that lncRNA-MEG3 only significantly defined the patients who needed splenectomy (AUC = 0.750, 95% CI: 0.599–0.867; *P* = *0.049*) with 100% sensitivity but 51.28% specificity at cutoff point 5.7(Table 4) (Fig. 2)

## Discussion

The main findings of this study showed that in patients with ITP, serum levels of lncRNA-MEG3, CXCL13, and NF-kB were significantly elevated, while miR-125a-5p was significantly decreased compared to healthy subjects. In addition, we found that these markers correlate well with the disease severity.

We found higher CXCL13 levels in ITP patients compared to controls and were associated with more severe bleeding, lower hemoglobin level, and need for transfusion. These results are similar to previous findings by Li et al. that CXCL13 protein was elevated in ITP patients and positively related to hemorrhage severity [16]. CXCL13, a B-cell chemokine produced mainly by mesenchymal lymphoid tissue organizer cells, follicular dendritic cells, and human T follicular helper cells, was found to be linked to a number of autoimmune diseases, including ITP [25]. CXCL13 is required for B-helper cell homing, antibody production, and a subset of T-helper cells to the lymphoid follicle that could explain it role in pathogenesis of ITP [26].

Our data showed decreased expression of miR-125a-5p in patients with ITP compared to healthy subjects. Furthermore, miR-125a-5p was inversely related to the CXCL13 level. Many miRNAs were differentially expressed (upregulated or downregulated) in patients with ITP [7]. In genome-wide miRNA expression study of three newly diagnosed adult patients with ITP and three healthy controls using microarray analysis showed that miR-125a-5p expression was down-regulated in patients with ITP [7]. CXCL13 was reported to be the target gene of miR-125a-5p [16]. Moreover, in transfected CD4 + T cells, CXCL13 level decreased with miR-125-5p mimic, but increased with miR-125a-5p suppressor [9]. Few in vivo studies measured miR-125a-5p in patients with ITP [7, 9]. Zhu et al. found that ITP patients have downregulated miR-125a-5p levels in Tregs, which contributes to Treg dysfunction and ITP pathogenesis [7].

NF-κb regulates multiple aspects of innate and adaptive immune responses. It is involved in the pathogenesis of many autoimmune conditions, such as rheumatoid arthritis, diabetes, multiple sclerosis, and inflammatory bowel disease [27]. However, the role of NF-κb in ITP needs to be elucidated. We found that patients with ITP had significantly higher NF-κB levels compared to healthy control, and NF-κB was negatively associated with platelet count. In contrast, Yu et al. reported that in patients with ITP, NF-κB level was lower than in control; however, their study included patients with recently diagnosed, chronic, and resistant ITP compared to our study, which included only newly diagnosed ITP patients [15].

Our results showed a significant negative correlation of NFκB with anti-Inflammatory MicroRNA, miR-125a-5p, and a positive correlation with CXCL13. Previous researches reported that NFκB pathway activation induced CXCL13-expression in lung cancer and osteosarcoma, promoting cell migration [28, 29]. Furthermore, several miRNAs play essential roles in the immune response via tuning NF-kB Pathway [30]. Also, it has been reported that miR-125a-p5 acts as an NFκB inhibitor upon the Toll-like receptor signaling (TLR) pathway’s activation, inhibiting erythroid differentiation in leukemia [30].

LncRNA is a protein-coding transcript that plays an important role in gene regulation [31]. In the current study, the lncRNA-MEG3 level was higher in patients with ITP than in healthy controls and was associated with lower platelet count and need for transfusion. Few studies investigated the role of lncRNA-MEG3 in ITP. Li et al. reported that lncRNA-MEG3 was significantly upregulated in CD4 + T cells of patients with ITP [9]. At the same time, its level reduced after therapy, suggesting the significant role of lncRNA-MEG3 in the pathogenesis of ITP [9]. Also, they found that lncRNA-MEG3 inhibits the miRNA-125a-5p expression and induces an immune imbalance of Treg/Th17 in ITP [9]; this explains the negative correlation between lncRNA-MEG3 and miR-125a we found in the current study.

We found that lncRNA-MEG3 had the best overall prediction of response to steroids and the need for splenectomy in ITP patients compared to miR-125a-5p, CXCL13, and NF-kB, which make lncRNA-MEG3 a potential prognostic marker in the management of ITP. Some studies have identified a significant relationship between lncRNA-MEG3 expression levels and treatment of ITP [9, 16]. Li et al. in vitro experiments found that dexamethasone decreased the lncRNA-MEG3 expression level of CD4 + T cells [9]. In addition, It has been shown that lncRNA-MEG3 levels are significantly increased in CD4 + T cells of patients with ITP, and that their levels are reduced after steroid treatment, which is explained by lncRNA-MEG3’s effect on Tregs/Th17 balance [9].

In the current study, CXCL13 was the most specific predictor of the severity of bleeding and the response to the steroids. In a study of children with ITP, Li et al. found elevated plasma CXCL13 and the concentration of which was reduced after treatment [16]. Moreover, they performed in vitro experiment in which dexamethasone was added to CD4 + T lymphocytes isolated from healthy volunteers. They found that dexamethasone decreased the CXCL13 level in a dose-dependent and time-dependent manner [16]. The higher specificity of CXCL13 in our study might be explained by its pivotal role in B1 cell homing and its vital role in the recruitment of B- cells and T- cell subsets [28].

This study has limitations to be mentioned. Firstly, Lack of functional studies. Although our results showed significant statistical correlation between lncRNA-MEG3, miR-125a-5P, and CXCL13 and NF-kb that could suggest a new molecular pathway in patients with ITP, it was lacking functional experiments. So, more research is needed for elucidating the role of lncRNA-MEG3, miR-125a-5p, CXCL13 and NF-kB in regulating the function of each other. Secondly, the levels of our markers were measured once in the beginning of the study. Because of limited resources we were not able to measure before and after treatment and further studies of the effect of steroid therapy and other immune therapies on serum level of these markers in patients with ITP are required. Thirdly, the patients came from one country; the results may not be representative of patients from other geographic areas. Further multicenter studies are needed to confirm the results in different populations.

In conclusion serum lncRNA-MEG3, CXCL13, and NF-kB increased in ITP patients and were negatively correlated with platelet count, while miR-125a-5p level decreased and was positively correlated with platelet count. They were good prognostic markers of response to steroid therapy with lncRNA-MEG3 had the best overall prediction of response to treatment. Also, lncRNA-MEG3 was the best predictor in identifying the patients need for splenectomy. In addition, the significant correlation between lncRNA-MEG3, CXCL13, miR-125a-5p, and NF-kB might suggest a novel molecular pathway in patients with ITP that starts with lncRNA-MEG3 overexpression that decreases miR-125a-5p expression, leading to increased CXCL13 probably through the NF-kB pathway which could be provide a new therapeutic target in the management of ITP.

## Data Availability

The data supporting the findings of this study are available from the corresponding author upon reasonable request.

## References

[1] Neunert CE (2017). Management of newly diagnosed immune thrombocytopenia: can we change outcomes?. Blood Adv.

[2] Song I, Kim J, Kwon K, Koo S, Jo D (2016). Expression of CD154 (CD40L) on stimulated T lymphocytes in patients with idopathic thrombocytopenic purpura. Hematology.

[3] Raphael I, Joern RR, Forsthuber TG (2020). Memory CD4+ T Cells in Immunity and Autoimmune Diseases. Cells.

[4] Kostic M, Zivkovic N, Cvetanovic A, Marjanović G (2020). CD4+ T cell phenotypes in the pathogenesis of immune thrombocytopenia. Cell Immunol.

[5] Ko NY, Chen LR, Chen KH (2020). The Role of Micro RNA and Long-Non-Coding RNA in Osteoporosis. Int J Mol Sci.

[6] Hirschberger S, Hinske LC, Kreth S (2018). MiRNAs: dynamic regulators of immune cell functions in inflammation and cancer. Cancer Lett.

[7] Zhu Y, Zhu H, Xie X, Zheng Z, Ling Y (2019). MicroRNA expression profile in Treg cells in the course of primary immune thrombocytopenia. J Investig Med.

[8] Xue H, Gao H, Xia H, Li S, Li N, Duan Y (2021). Prognostic significance of long non coding maternally expressed gene 3 in pediatric acute myeloid leukemia. Med (Balt).

[9] Li JQ, Hu SY, Wang ZY, Lin J, Jian S, Dong YC (2016). Long non-coding RNA MEG3 inhibits microRNA-125a-5p expression and induces immune imbalance of Treg/Th17 in immune thrombocytopenic purpura. Biomed Pharmacother.

[10] Shi Y, Wang Y, Luan W, Wang P, Tao T, Zhang J (2014). Long non-coding RNA H19 promotes glioma cell invasion by deriving miR-675. PLoS ONE.

[11] Wang P, Liu YH, Yao YL, Li Z, Li ZQ, Ma J (2015). Long non-coding RNA CASC2 suppresses malignancy in human gliomas by miR-21. Cell Signal.

[12] Lee HT, Shiao YM, Wu TH, Chen WS, Hsu YH, Tsai SF (2010). Serum BLC/CXCL13 concentrations and renal expression of CXCL13/CXCR5 in patients with systemic lupus erythematosus and lupus nephritis. J Rheumatol.

[13] Workel HH, Lubbers JM, Arnold R, Prins TM, van der Vlies P, de Lange K (2019). A Transcriptionally Distinct CXCL13+CD103+CD8+ T-cell Population Is Associated with B-cell Recruitment and Neoantigen Load in Human Cancer. Cancer Immunol Res.

[14] Miraghazadeh B, Cook MC (2018). Nuclear Factor-kappaB in Autoimmunity: Man and Mouse. Front Immunol.

[15] Yu J, Hua M, Zhao X, Wang R, Zhong C, Zhang C (2018). NF-κB-94ins/del ATTG Genotype Contributes to the Susceptibility and Imbalanced Th17 Cells in Patients with Immune Thrombocytopenia. J Immunol Res.

[16] Li JQ, Hu SY, Wang ZY, Lin J, Jian S, Dong YC (2015). MicroRNA-125-5p targeted CXCL13: a potential biomarker associated with immune thrombocytopenia. Am J Transl Res.

[17] Li J, Bai J, Tuerdi N, Liu K (2022). Long non-coding RNA MEG3 promotes tumor necrosis factor-alpha induced oxidative stress and apoptosis in interstitial cells of cajal via targeting the microRNA-21 /I-kappa-B-kinase beta axis. Bioengineered.

[18] Kim SW, Ramasamy K, Bouamar H, Lin AP, Jiang D, Aguiar RC (2012). MicroRNAs miR-125a and miR-125b constitutively activate the NF-κB pathway by targeting the tumor necrosis factor alpha-induced protein 3 (TNFAIP3, A20). Proc Natl Acad Sci USA.

[19] Rodeghiero F, Michel M, Gernsheimer T, Ruggeri M, Blanchette V, Bussel JB (2013). Standardization of bleeding assessment in immune thrombocytopenia: report from the International Working Group. Blood.

[20] Provan D, Arnold DM, Bussel JB, Chong BH, Cooper N, Gernsheimer T (2019). Updated international consensus report on the investigation and management of primary immune thrombocytopenia. Blood Adv.

[21] Shaker O, Alhelf M, Morcos G, Elsharkawy A (2017). miRNA-101-1 and miRNA-221 expressions and their polymorphisms as biomarkers for early diagnosis of hepatocellular carcinoma. Infect Genet Evol.

[22] Shaker O, Mahfouz H, Salama A, Medhat E (2020). Long Non-Coding HULC and miRNA-372 as Diagnostic Biomarkers in Hepatocellular Carcinoma. Rep. Biochem Mol Biol.

[23] Rao X, Huang X, Zhou Z, Lin X (2013). An improvement of the 2ˆ(-delta delta CT) method for quantitative real-time polymerase chain reaction data analysis. Biostat Bioinforma Biomath.

[24] Fang L, Liu J, Wan L, Zhu F, Tan B, Zhang P. [Xinfeng capsule improves hypercoagulative state by inhibiting miR-155/NF-κB signaling pathway in patients with active ankylosing spondylitis]. Xi Bao Yu Fen Zi Mian Yi Xue Za Zhi. 2016;32:1094–8. Chinese. 27412942

[25] Pan Z, Zhu T, Liu Y, Zhang N (2022). Role of the CXCL13/CXCR5 Axis in Autoimmune Diseases. Front Immunol.

[26] Sáez de Guinoa J, Barrio L, Mellado M, Carrasco YR (2011). CXCL13/CXCR5 signaling enhances BCR-triggered B-cell activation by shaping cell dynamics. Blood.

[27] Barnabei L, Laplantine E, Mbongo W, Rieux-Laucat F, Weil R (2021). NF-κB: At the Borders of Autoimmunity and Inflammation. Front Immunol.

[28] Chao CC, Lee WF, Wang SW, Chen PC, Yamamoto A, Chang TM (2021). CXC chemokine ligand-13 promotes metastasis via CXCR5-dependent signaling pathway in non-small cell lung cancer. J Cell Mol Med.

[29] Liu JF, Lee CW, Lin CY, Chao CC, Chang TM, Han CK (2020). CXCL13/CXCR5 Interaction Facilitates VCAM-1-Dependent Migration in Human Osteosarcoma. Int J Mol Sci.

[30] Tahamtan A, Teymoori-Rad M, Nakstad B, Salimi V (2018). Anti-Inflammatory MicroRNAs and Their Potential for Inflammatory Diseases Treatment. Front Immunol.

[31] Marchese FP, Raimondi I, Huarte M (2017). The multidimensional mechanisms of long noncoding RNA function. Genome Biol.

